# Ectopic liver tissue on the gallbladder wall with independent vascular supply: a case report

**DOI:** 10.3389/fmed.2026.1923004

**Published:** 2026-08-17

**Authors:** Longfei Wu, Yuan Tian, Congyan Chen, Yibo Kang, Xin Wang

**Affiliations:** 1 Department of General Surgery, West China Hospital, West China Xiamen Hospital, Sichuan University, Xiamen, Fujian, China; 2 Department of Radiology, West China Hospital, West China Xiamen Hospital, Sichuan University, Xiamen, Fujian, China; 3 Division of Pancreatic Surgery, Department of General Surgery, West China Hospital of Sichuan University, Chengdu, China

**Keywords:** case report, cholelithiasis, ectopic liver, indocyanine green fluorescence-guided, laparoscopic cholecystectomy

## Abstract

Ectopic liver (EL) is hepatic tissue with no parenchymal or ductal continuity with the native liver, even though it may receive an aberrant arterial supply, including a branch arising from the hepatic arterial system. While the gallbladder serosa represents the most frequently reported site for this anomaly, cases presenting with a clearly identifiable, independent vascular supply remain uncommon in clinical practice, often posing a diagnostic challenge during routine hepatobiliary evaluations. A 65-year-old male presented with a 2-year history of intermittent right upper quadrant abdominal pain. Preoperative non-contrast computed tomography (CT) demonstrated a hyperdense lesion within the gallbladder measuring approximately 2.7 × 2.1 cm, consistent with cholelithiasis. Notably, the imaging also revealed an exophytic nodular measuring approximately 1.2 × 0.8 cm firmly attached to the serosal surface of the anterior wall near the fundus. The patient subsequently underwent indocyanine green (ICG) fluorescence-guided laparoscopic cholecystectomy. Intraoperatively, a small, well-circumscribed, liver-like nodule was identified on the gallbladder wall. Under real-time near-infrared fluorescence, the nodule retained ICG and fluoresced bright green, identical to the native liver. The anomaly was completely excised en bloc with the gallbladder to prevent tissue fragmentation. Subsequent histopathological evaluation confirmed normal hepatic lobules and portal triads, establishing a definitive diagnosis of true ectopic liver tissue. Distinguishing EL from accessory hepatic lobes is clinically crucial, our case confirms true EL through complete anatomical separation and independent vascular pedicle. This case underscores the importance of meticulous intraoperative exploration and highlights the utility of ICG fluorescence in ensuring clear visualization during surgical resection.

## Introduction

Ectopic liver refers to hepatic tissue situated outside the main liver without direct parenchymal or biliary ductal continuity (1–3). It is an uncommon developmental anomaly, with a reported incidence of approximately 0.24–0.47% in surgical and autopsy series (4). Ectopic hepatic tissue may be present in a variety of distinct anatomical locations, such as at the gastrohepatic ligament, umbilical cord, adrenal glands, pancreas, pylorus, diaphragm, splenic capsule (5–7). Rare cases have also been described in extra-abdominal sites such as the thoracic cavity and esophagus (8, 9). Among these, the gallbladder is the most commonly reported site of ectopic liver. In these cases, the ectopic tissue is typically located on the serosal surface, most frequently involving the fundus and body. Less commonly, it may be identified within the mesentery, and only rarely within the gallbladder wall or lumen (10).

Although the gallbladder represents the most common site of ectopic liver, cases with a distinctly identifiable independent vascular supply remain exceedingly rare. Clinically, ectopic liver tissue has been reported in case literature to be associated with rare complications such as torsion, hemorrhage, or malignant transformation into hepatocellular carcinoma (11–13). In this report, we describe a rare case of ectopic liver attached to the gallbladder with a distinctly identifiable independent vascular supply. Although preoperatively suspected as a gallbladder wall mass on CT, the lesion was definitive identified, demarcated, and resected under indocyanine green (ICG) fluorescence-guided laparoscopic cholecystectomy.

### Case presentation

A 65-year-old male presented with a history of intermittent right upper quadrant pain for 2 years. The detailed clinical timeline, diagnostic workup, surgical intervention, and follow-up course are summarized in Table 1. The pain was characteristically exacerbated by the ingestion of greasy or fatty foods and was occasionally accompanied by referred pain to the right shoulder and back. There were no associated symptoms of fever, chills, or jaundice. While the patient had managed these symptoms conservatively for 2 years, the frequency of pain episodes had slightly increased prior to admission. However, he was admitted electively during a temporary asymptomatic window and presented with no ongoing biliary colic or acute cholecystitis at the time of admission and evaluation. The patient sought medical attention for further evaluation and definitive management of suspected symptomatic cholelithiasis. The patient had no significant prior medical history of hepatitis, cirrhosis, or abdominal surgery. He denied any history of smoking or chronic alcohol consumption. There was no known family history of gastrointestinal malignancies, hepatocellular carcinoma, or hereditary hepatobiliary diseases.

**Table 1:** Chronological timeline of clinical events, diagnostic evaluation, surgical intervention, and follow-up.

Timeline	Clinical events, diagnostics, and interventions
2 years prior to admission	Onset of intermittent right upper quadrant abdominal pain, exacerbated by fatty foods. Symptoms managed conservatively over 2 years with a slight increase in frequency prior to admission.
Day 3	Patient presented electively during an asymptomatic window for evaluation. Abdominal ultrasound and unenhanced CT revealed cholelithiasis (2.7 × 2.1 cm hyperdense lesion) and a 1.2 × 0.8 cm exophytic serosal nodule near the gallbladder fundus. Preoperative CA19-9 measured at 35.5 U/mL.
Day 1	Intravenous administration of ICG (0.05 mg/kg) 12 h prior to scheduled surgery for intraoperative biliary mapping.
Day 0	Surgical Intervention: Underwent ICG fluorescence-guided laparoscopic cholecystectomy. Real-time NIR fluorescence confirmed vivid green retention in the nodule. Precise white-light dissection identified an independent feeding artery originating from the RHA. En bloc resection performed.
Day 2	Uneventful postoperative recovery; patient discharged home in stable condition. Postoperative histopathology confirmed a true ectopic liver containing normal portal triads.
Month 3	Follow-up: Patient remained completely asymptomatic with well-healed surgical wounds. Liver function panel was normal, and serum CA19-9 decreased to 28.0 U/mL (Roche Cobas e801 platform).

CA19-9, carbohydrate antigen 19–9; CT, computed tomography; ICG, indocyanine green; NIR, near-infrared; RHA, right hepatic artery.

On admission, the patient’s vital signs were stable: body temperature 36.6 °C, pulse 74 bpm, respiratory rate 18 breaths/min, and blood pressure 128/82 mmHg. General Status: The patient was conscious and appeared in good nutritional status with no signs of anemia or jaundice. Abdominal Examination: The abdomen was flat and symmetrical without visible superficial veins or surgical scars. Upon palpation, the abdomen was soft; no deep tenderness, rebound tenderness, or muscle rigidity was elicited in the right upper quadrant. Murphy’s sign was negative. No abnormal masses, including the liver or spleen, were palpable under the costal margins. Laboratory results, encompassing liver function assessments and tumor markers such as alpha-fetoprotein (AFP), were within normal reference ranges. The carbohydrate antigen 19–9 (CA19-9) level was measured at 35.5 U/mL, exceeding the reference threshold of ≤30 U/mL.

**Figure 1:**
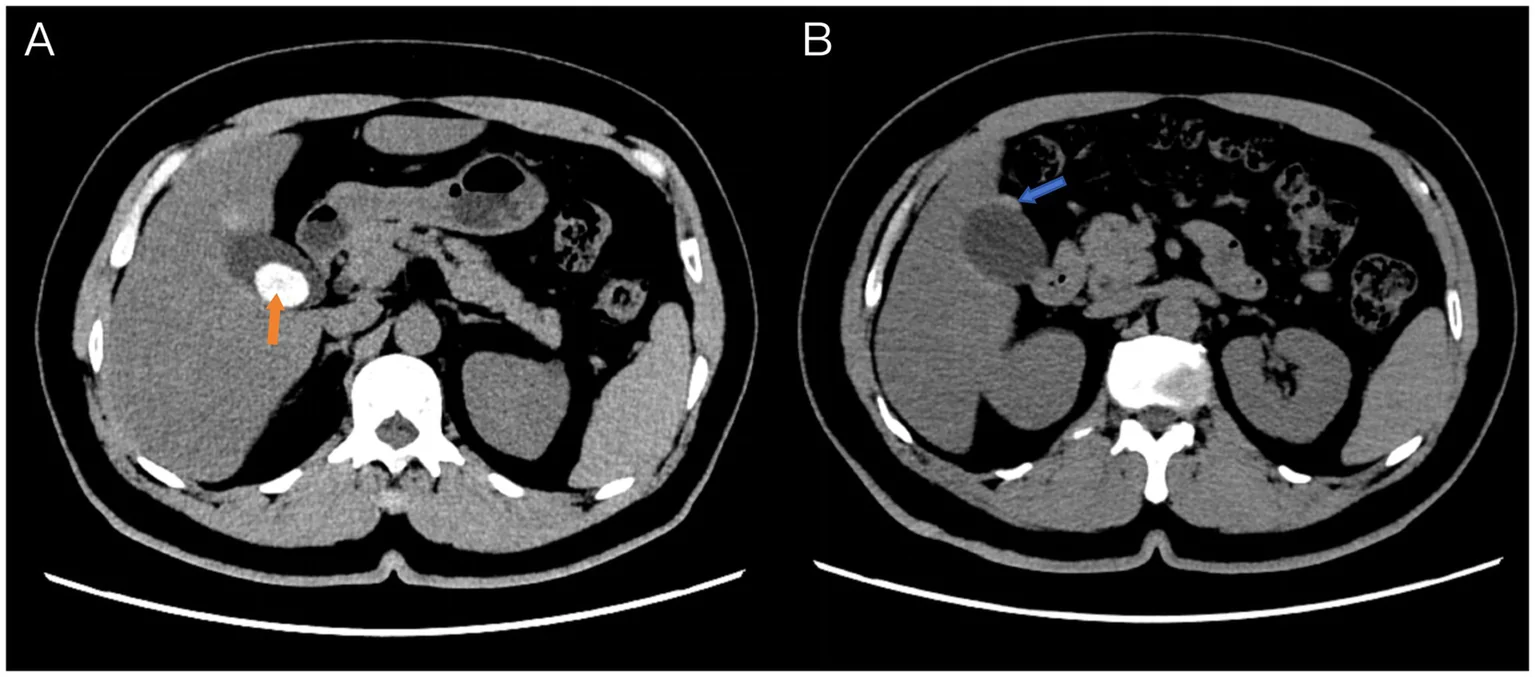
Preoperative computed tomography (CT) findings of the gallbladder and an exophytic nodular **(A)** A hyperdense lesion (orange arrow) is noted within the gallbladder lumen, consistent with a gallstone. **(B)** Unenhanced CT image shows an exophytic nodular (blue arrow) attached to the serosal surface of the anterior wall near the fundus, exhibiting a homogeneous density isodense to the native liver.

Preoperative abdominal ultrasonography demonstrated a hyperechoic lesion within the gallbladder measuring approximately 2.5 × 1.9 cm, exhibiting posterior acoustic shadowing and gravity-dependent mobility, consistent with cholelithiasis. Non-contrast computed tomography (CT) demonstrated a hyperdense lesion within the gallbladder measuring approximately 2.7 × 2.1 cm (Figure 1A). These minor dimension discrepancies were modality-dependent. In addition, an exophytic nodular measuring approximately 1.2 × 0.8 cm attached to the serosal surface of the anterior wall near the fundus (Figure 1B). On unenhanced CT, the lesion exhibited a homogeneous density, which was isodense to the adjacent hepatic parenchyma.

Diagnostic reasoning and differential diagnosis: Preoperatively, the identification of a gallbladder-bound mass presents a significant diagnostic challenge. Given the 1.2 cm exophytic serosal nodule detected on CT, the differential diagnosis included: (1) accessory hepatic lobe, which typically maintains direct parenchymal or ductal continuity with the main liver bed; (2) exophytic gallbladder polyp or adenomyomatosis, which usually projects into the luminal mucosal layer rather than the outer serosa; (3) primary or metastatic gallbladder neoplasm, which often presents with ill-defined margins or adjacent invasive features.

The preoperative working diagnosis was cholelithiasis accompanied by an exophytic nodule. The patient underwent ICG fluorescence-guided laparoscopic cholecystectomy. ICG produced by Dandong Yichuang Pharmaceutical Co., Ltd., was administered intravenously (IV) via the peripheral vein at a dose of 0.05 mg/kg, approximately 12 h prior to surgery, achieving cholangiography (14). Platform & Mode: OptoMedic Stellar 4K 3D all-in-one endoscopic imaging system, utilizing 4K Standard Fluorescence Overlay Mode for synchronous fusion of white-light and NIR fluorescence signals. Following the intravenous administration of ICG, a near-infrared fluorescence imaging system was employed to enhance the visualization of the biliary anatomy and the suspected lesion.

**Figure 2:**
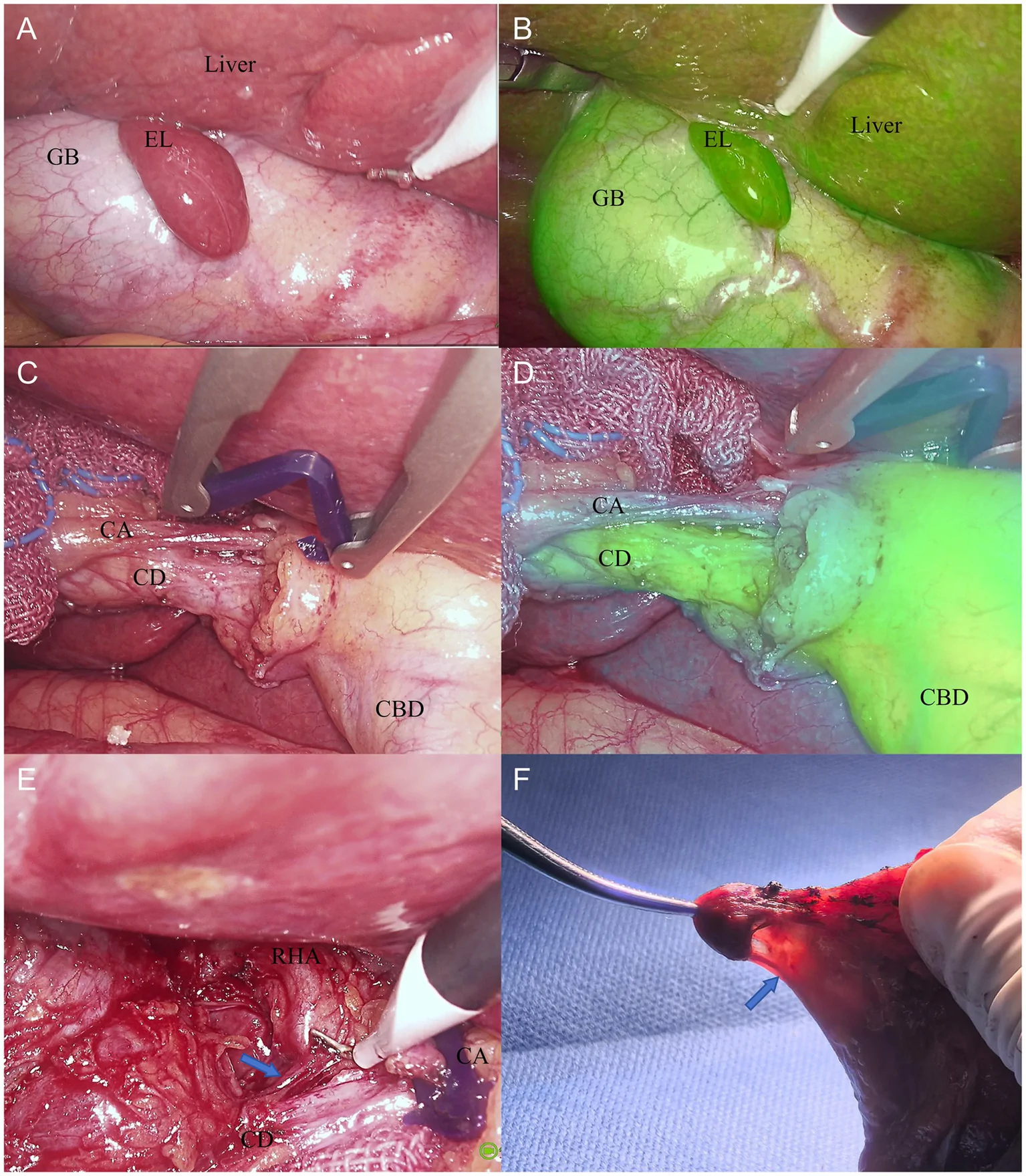
Intraoperative findings during indocyanine green fluorescence-guided laparoscopic cholecystectomy. GB, gallbladder; EL, ectopic liver; CD, cystic duct; CA, cystic artery; CBD, common bile duct. **(A)** White light visualization shows the GB with EL attached to the serosal surface of the anterior wall near the fundus. **(B)** Corresponding near-infrared (NIR) fluorescence view demonstrating intense, uniform ICG retention within the EL (blue arrow), displaying a vivid green fluorescence signal identical in intensity to the adjacent native liver parenchyma. **(C)** High-magnification white-light view showing Calot’s triangle, identifying the CD, CA, and CBD. **(D)** Intraoperative near-infrared fluorescence-overlay mode displaying strong biliary fluorescence signal in the CD and CBD, alongside the CA visualised under the white-light overlay component. **(E)** High-magnification view reveals a discrete artery (blue arrow) supplying the ectopic liver, originating separately from the cystic artery. RHA, right hepatic artery. **(F)** Close-up view of the EL highlights an independent vascular pedicle (blue arrow).

Intraoperatively, a small exophytic nodular lesion measuring approximately 1.2 × 0.8 cm was identified arising from the serosal surface of the anterior wall near the fundus (Figures 2A,B). High-magnification white-light and NIR fluorescence view showing Calot’s triangle, identifying the cystic duct, cystic artery and common bile duct (Figures 2C,D). A discrete feeding artery supplying the lesion was noted along the gallbladder wall. This vessel ran independently of the cystic artery branch, with both vessels traced back as branches originating from the right hepatic artery (Figures 2E,F). Intraoperatively, after standard identification and clip-ligation of the main cystic artery, the nodule demonstrated no apparent ischemic changes or discoloration, raising strong clinical suspicion of an alternative or accessory arterial supply. Subsequent meticulous dissection along the Calot’s triangle revealed a distinct secondary feeding vessel originating separately from the right hepatic artery (RHA). Upon selective ligation and division of this vessel, the ectopic hepatic nodule immediately exhibited clear ischemic cyanosis (discoloration), confirming that this RHA-derived vessel provided the primary, independent arterial nourishment to the ectopic liver tissue (Figure 2F).

**Figure 3:**
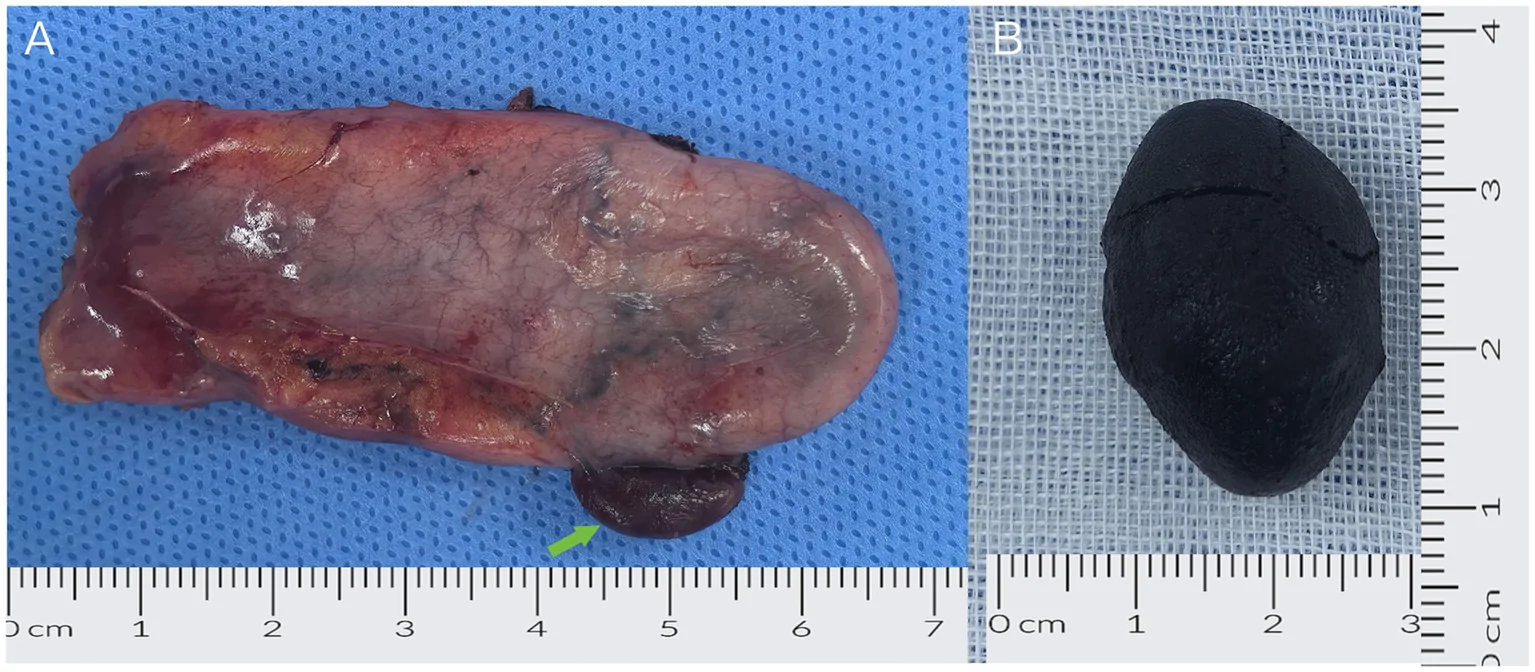
Gross appearance of the resected gallbladder and ectopic liver. **(A)** External view of the gallbladder shows an exophytic nodular (green arrow) measuring (1.2 cm) attached to the serosal surface of the anterior wall near the fundus. **(B)** Opened gallbladder specimen reveals a large, solitary greenish-black gallstone measuring 2.7 × 2.1 cm. The stone exhibits a characteristic internal core upon sectioning, with a brittle texture.

The vessel was carefully ligated, and the exophytic nodular lesion was resected en bloc with the gallbladder. No direct anatomical continuity with the native liver was identified. On gross examination, an oval, greenish-black gallstone measuring approximately 2.7 × 2.1 cm was identified within the gallbladder lumen (Figure 3A). The stone exhibited a well-defined concentric lamellated architecture, suggestive of chronic deposition (Figure 3B).

**Figure 4:**
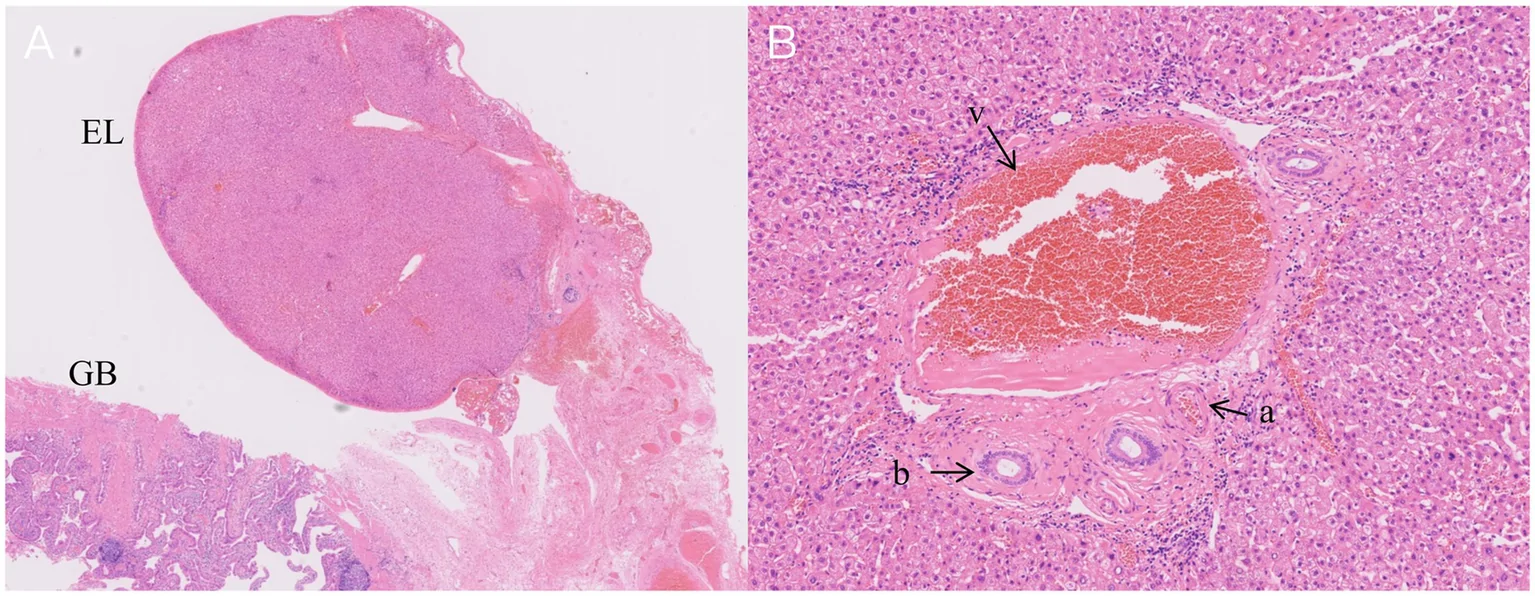
Histopathological examination of the ectopic liver tissue. **(A)** Low-power histological overview displaying well-differentiated hepatic parenchyma (H&E staining; original magnification ×40; scale bar = 250 μm). GB, gallbladder; EL, ectopic liver. **(B)** High-power view clearly demonstrating a complete portal triad structure (H&E staining; original magnification ×200; scale bar = 40 μm). a, interlobular artery; v, interlobular vein; b, interlobular bile duct.

Histopathological examination revealed normal hepatic architecture (Figure 4). Microscopic evaluation of the resected tissue subsequently confirmed the presence of a complete triad structure (interlobular artery, interlobular vein, and interlobular bile duct) within the classic portal triads of the ectopic liver tissue. No evidence of dysplasia, cirrhosis, or malignancy was identified. The findings confirmed the diagnosis of ectopic liver. Further immunohistochemical (IHC) staining was not performed on the archived paraffin blocks per institutional diagnostic policy and the patient’s informed preference, as routine H&E staining provided unambiguous morphological criteria for benign hepatic tissue without cytological atypia.

The postoperative course was uneventful and the patient was discharged on postoperative day 2. Follow-up was conducted via outpatient clinical visits and laboratory testing at 3 months postoperatively. The patient reported complete resolution of abdominal symptoms, normal physical examination, well-healed surgical wounds without incisional hernia or infection, no readmissions, and a completely normal liver function panel. Laboratory re-evaluation revealed a completely normal liver function panel and a postoperative serum CA19-9 level of 28.0 U/mL (quantified via electrochemiluminescence immunoassay on the Roche Cobas e801 platform; institutional reference threshold: <30.0 U/mL).

### Patient perspective

The patient expressed high satisfaction with the complete relief of chronic pain and reassurance regarding the benign nature of the nodule.

## Discussion

Ectopic liver is a rare congenital anomaly thought to arise from aberrant migration or displacement of hepatic tissue during embryological development (1). Several mechanisms have been proposed, including sequestration of a fragment of the hepatic diverticulum or aberrant migration of hepatocyte precursor cells (15, 16). The gallbladder is the most common location, likely due to its close embryological relationship with the liver (2, 17). According to the classic anatomical classification proposed by Collan et al. (1), ectopic liver tissue is categorized into four distinct types: Type I (true ectopic liver, completely separated from the main liver without parenchymal or ductal connection), Type II (microscopic ectopic liver), Type III (large accessory liver lobe with a direct connection), and Type IV (small accessory liver lobe connected via a tissue bridge). In the present case, the absence of anatomical continuity and the presence of an independent vascular pedicle, strongly support the diagnosis of ectopic liver.

While ectopic liver tissue typically recruits local vascular supply from adjacent structures (such as cystic or mesenteric branches), our intraoperative exploration demonstrated a distinct anatomical variant featuring an discrete feeding artery derived from the main right hepatic artery. Most published reports lack detailed characterization of the vascular supply of ectopic liver tissue. Based on available reports, three principal vascular patterns have been described in gallbladder-associated cases: (1) an arterial branch originating from the cystic artery (18); (2) a vascular pedicle arising from the hepatic parenchyma (19); and (3) vascular structures running within a mesentery connecting the native liver to the ectopic hepatic tissue. Distinct from previously documented cases, the ectopic liver nodule in our patient was nourished by a separate arterial pedicle originating from the right hepatic artery rather than the cystic artery. Puledda et al. (20) utilized ICG for the intraoperative identification of gallbladder heterotopic liver, but the tissue relied on local diffusion without a major macroscopically tracked vascular stalk. Such vascularization supports the hypothesis that some ectopic liver tissues may develop as accessory hepatic buds with partial autonomy. Reports describing well-defined arterial and/or venous supply are scarce, making this case noteworthy.

Although most cases are asymptomatic and are typically diagnosed incidentally during laparotomy, laparoscopy, or autopsy, ectopic liver should not be regarded as a purely incidental finding (3, 21). Both benign and malignant lesions may arise in ectopic liver tissue. Ectopic liver, similar to native hepatic parenchyma, may undergo pathological changes such as steatosis, hemosiderosis, cholestasis, cirrhosis, hepatitis, and malignant transformation into hepatocellular carcinoma (11). Previous studies have established that ectopic liver tissue can harbor various benign hepatic lesions, such as cavernous hemangiomas, focal nodular hyperplasia, and hepatic cell adenomas (22–27). In contrast, hepatocellular carcinoma (HCC) represents the most frequently reported malignant transformation in ectopic liver tissue, with a substantial proportion of cases reported in Japan (12, 13, 28–31). Kubota et al. (7). reported a case of a 56-year-old man presenting with a pancreatic tail mass, which was confirmed postoperatively as hepatocellular carcinoma arising from ectopic liver tissue on histopathological examination. Despite the mild increment of CA19-9 noted here, mild elevations or high-normal values of CA19-9 are common and non-specific in cholelithiasis due to chronic inflammation.

The malignant transformation of ectopic liver tissue has been attributed to multiple mechanisms, including impaired vascular and biliary drainage, metabolic dysfunction, and chronic inflammatory stimulation, which collectively may establish a microenvironment conducive to hepatocarcinogenesis (10, 12, 28). Our present case exhibited no such structural or functional impairment at the time of resection. Although literature consisting of retrospective case reports suggests that ectopic liver tissue has been associated with chronic inflammatory stress or, in rare instances, malignant transformation into HCC, this risk must be contextualized. For EL situated specifically on the gallbladder wall, routine laparoscopic cholecystectomy achieves complete en bloc resection, effectively eliminating any long-term oncological threat, unlike intra-abdominal or retroperitoneal EL sites that might escape routine detection.

This study has several limitations. First, as a single case report, the findings regarding the unique vascular pedicle cannot be generalized to all gallbladder-associated EL cases. RHA as the vessel of origin was based solely on high-definition intraoperative visual dissection without preoperative CT angiography (CTA) or formal digital subtraction angiography (DSA). Second, preoperative evaluation relied on non-contrast CT due to the initial suspicion of routine gallstones, which precluded retrospective multi-phase CT angiography reconstruction. Lastly, although H&E staining demonstrated definitive and classic benign hepatic histology, specialized immunohistochemical (IHC) characterization (such as HepPar-1 or CK7/19) was not performed because the patient’s family declined additional diagnostic testing. Nevertheless, the combination of distinct intraoperative morphology, clear H&E features, and clear ICG fluorescence characteristics strongly supports the diagnosis. While the patient achieved complete symptom relief and demonstrated normal laboratory findings postoperatively, the follow-up period (3 months) remains relatively short. Long-term surveillance data regarding potential delayed systemic effects or localized recurrence are naturally absent.

From a surgical perspective, the presence of an independent vascular pedicle in ectopic liver tissue represents a potential source of intraoperative bleeding if not carefully identified. This underscores the importance of recognizing rare vascular variants during routine cholecystectomy and supports the value of preoperative imaging and intraoperative vigilance in preventing iatrogenic injury.

## Conclusion

This case describes ectopic liver tissue attached to the gallbladder wall with a discrete feeding vessel. In this patient, near-infrared indocyanine green (ICG) fluorescence provided essential real-time visualization for confirming the functional hepatic parenchyma of the ectopic nodule and delineating the extrahepatic biliary anatomy, while precise white-light dissection enabled the identification and ligation of its independent feeding vessel along the gallbladder serosa during laparoscopic cholecystectomy. Recognition of this anatomical variation and the combined application of fluorescence and white-light visualization are clinically meaningful for maintaining intraoperative surgical safety.

## Data Availability

The original contributions presented in the study are included in the article/supplementary material, further inquiries can be directed to the corresponding author.
